# Evolutionary and genetic analysis of the VP2 gene of canine parvovirus

**DOI:** 10.1186/s12864-017-3935-8

**Published:** 2017-07-17

**Authors:** Gairu Li, Senlin Ji, Xiaofeng Zhai, Yuxiang Zhang, Jie Liu, Mengyan Zhu, Jiyong Zhou, Shuo Su

**Affiliations:** Jiangsu Engineering Laboratory of Animal Immunology, Institute of Immunology and College of Veterinary Medicine, Nanjing Agricultural University, Nanjing, China

**Keywords:** Canine parvovirus type 2, VP2, Phylogenetic analysis, Evolution, Codon usage

## Abstract

**Background:**

Canine parvovirus (CPV) type 2 emerged in 1978 in the USA and quickly spread among dog populations all over the world with high morbidity. Although CPV is a DNA virus, its genomic substitution rate is similar to some RNA viruses. Therefore, it is important to trace the evolution of CPV to monitor the appearance of mutations that might affect vaccine effectiveness.

**Results:**

Our analysis shows that the VP2 genes of CPV isolated from 1979 to 2016 are divided into six groups: GI, GII, GIII, GIV, GV, and GVI. Amino acid mutation analysis revealed several undiscovered important mutation sites: F267Y, Y324I, and T440A. Of note, the evolutionary rate of the CPV VP2 gene from Asia and Europe decreased. Codon usage analysis showed that the VP2 gene of CPV exhibits high bias with an ENC ranging from 34.93 to 36.7. Furthermore, we demonstrate that natural selection plays a major role compared to mutation pressure driving CPV evolution.

**Conclusions:**

There are few studies on the codon usage of CPV. Here, we comprehensively studied the genetic evolution, codon usage pattern, and evolutionary characterization of the VP2 gene of CPV. The novel findings revealing the evolutionary process of CPV will greatly serve future CPV research.

**Electronic supplementary material:**

The online version of this article (doi:10.1186/s12864-017-3935-8) contains supplementary material, which is available to authorized users.

## Background

Canine parvovirus (CPV) belongs to the genus *Protoparvovirus* and causes severe intestinal disease and leukopenia among carnivores, especially in canines and felines [1, 2]. CPV is a non-enveloped DNA virus with an approximately 5000-nucleotide, single-stranded DNA genome including two open reading frames (ORFs). The first ORF encodes two non-structural proteins (NS1 and NS2) and the second ORF encodes two structural proteins (VP1 and VP2) [3]. VP2 is the most abundant structural protein, accounting for 90% of the viral capsid, and is able to self-assemble to make virus like particles (VLPs) [4]. VP2 is a major antigenic determinant and plays a critical role in determining viral tissue tropism and host range [5, 6]. Notably, only a few amino acid substitutions in its sequence can alter relevant biological characteristics of the virus [7]. NS1, is a pleiotropic nuclear phosphoprotein that is important for viral replication and is responsible for inducing apoptosis [8, 9]. Canine parvovirus (also known as CPV type 2) emerged in 1978 in the USA and quickly spread among dog populations all over the world with high morbidity [10]. The virus experiences continuous genetic variation. CPV-2a, CPV-2b, and CPV-2c are the current three main antigenic variants of CPV [11]. Amino-acid substitutions at specific VP2 residues are the basis for the classification of CPV type 2 viruses into variants CPV-2a, CPV-2b and CPV-2c [12, 13].

Group of codons that encode the same amino acid are generally referred to as ‘synonymous’ codons, although their corresponding tRNAs may be different from their relative abundance in cells and the ribosome recognition speed. Notably, the usage of synonymous codons is a non-random selection process. Some codons are used more often than others [14, 15], a phenomenon referred to as “codon usage bias” that can be found in numerous species, such as prokaryotes, eukaryotes, and viruses [16]. Previous studies revealed that codon usage patterns are influenced by natural selection and mutation bias [17, 18]. The differential usage of synonymous codons (among other aspects of genome evolution) might be crucial to the understanding of viral biology, especially the interplay between viruses and the immune response. Genome-wide mutational pressure is the most important factor shaping patterns of codon usage bias in DNA viruses [19, 20]. Thus, understanding the codon usage of viruses can provide information about the mechanisms driving molecular evolution and expand our understanding of the regulation of viral gene expression. This will ultimately improve live attenuated vaccine antigenicity due to more efficient viral gene expression.

Compared with RNA viruses, DNA viruses have a lower mutation rate, thus, until now there has been very little research focusing on codon usage bias of animal DNA viruses. Of note, although CPV is a DNA virus, the genomic substitution rate of CPV is approximate to 10^−4^ per site per year, similar to RNA viruses [21]. Previous studies showed that mutation pressure constrained by nucleotide composition and natural selection are two decisive forces driving the codon usage of DNA virus. To describe the genetic features of the VP2 gene of CPV, we analyzed in detail the genetic evolution, the codon usage pattern, and the evolutionary patterns of VP2 of CPV.

## Results

### Phylogenetic analysis and amino acid mutant site analysis

**Fig. 1:**
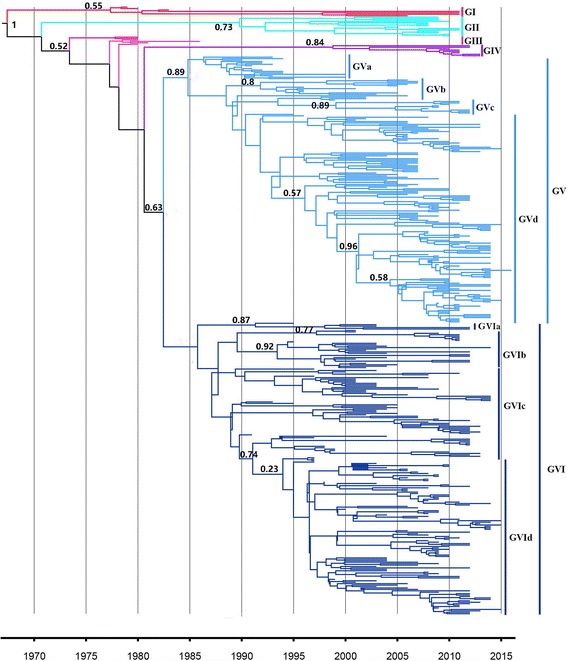
Phylogenetic and temporal relationships of 424 CPV sequences of the VP2 gene (1755 bp) worldwide were reconstructed using BEAST. Values of Bayesian posterior probability >0.5 are displayed. GI is represented in *red*, GII in *green*, GIII in *pink*, GIV in *purple*, GV in *cyan*, and GVI in *blue*

To analyze the genetic diversity and evolution dynamics of CPV, we collected 424 full-length VP2 sequences of CPV obtained over a period of 38 years (from 1979 to 2016) from 21 countries that reported CPV epidemics and the phylogeny of the full-length VP2 gene (1755 base pairs) was reconstructed (Fig. 1). Based on the phylogeny, nucleotide identity (less than 99.7%) and Bayesian posterior probabilities, we identified six clades: GI to GVI. Group GI includes the earliest two CPV sequences from the USA from 1979 and 1980 and the earliest sequences from China from 1983; it also includes several recent origin CPV-2 isolates, suggesting that the early CPV-2 strain has re-emerged in canine populations in India and China. Sequences belonging to group GII are mainly from China, Thailand, and India from 2008 to 2011. Group GIII mainly consists of the early CPV-2a sequences from epidemics in Brazil in 1980, while group GIV mainly consists of sequences from the USA.

Sequences in group GV can be divided into four subgroups: GVa, GVb, GVc and GVd. GVa comprises CPV-2a strains from an epidemic in Brazil. Sequences in GVb and GVd have mainly circulated in Asia from 1995 to date, including China, Japan, India, South Korea, et. These sequences include different genotypes of CPV in these countries. Sequences in GVc are mainly from the USA, except for one from China. Similarly to group GV, group GVI can also be divided into four subgroups: GVIa, GVIb, GVIc and GVId. GVIa and GVIb mainly consist of sequences belonging to CPV-2a. GVIc is mainly composed of genotype CPV-2b sequences and almost all CPV-2c sequences form another independent subgroup, group GVId. Overall, early CPV 2a sequences mainly belong to Group GI and GII. CPV 2a is classified into clades GIII, GVd, GVIa and GVIb. CPV 2b sequences cluster with clades of GVd, and GVIc, while CPV-2c is mainly clusters with the GVId group in this study.

Interestingly, analysis of the amino acid alignments (Table 1) revealed universal substitution of amino acid sites: K80R, K93 N, V103A, D323N, N564S, and A568G. In addition, mutant sites discovered in special genotype: M87 L, I101T, A300G, and D305Y in CPV-2a; N426D substitution in CPV-2b in 1984; and N426E substitution in CPV-2c. Moreover, new CPV-2a and CPV-2b carrying a S297A substitution were identified. Other potential important substitutions were also found in CPV isolates: F267Y, Y324I, and T440A (Table 1). The detailed numbers of the three sites of the 424 sequences are listed in the Additional file 1: Table S1. Additionally, a Q370R change was found in some CPV-2c strains isolated from China.

**Table 1:** Mutation sites and amino acid mutations of the CPV VP2 gene

Accession number and virus name	Virus type	Amino Acid VP2									
		87	101	300	305	297	267	324	440	370	426
EU659116/CPV-5.us.79/1979/USA	Origin CPV type 2	M	I	A	D	S	F	Y	T	Q	N
GU212791/VAC-P vanguard/2009/Thailand		M	I	A	D	S	F	Y	T	Q	N
DQ340408/BR154–80/1980/Brazil	CPV-2a	L	T	G	Y	S	F	Y	T	Q	N
JX120178/CPV-GZ/2010/China		L	T	G	Y	A	Y	I	A	Q	N
KM457132/ UY245/2010/ Uruguay		L	T	G	Y	A	Y	I	A	Q	N
JQ996151/ YAZA2/2010/China		L	T	G	Y	A	Y	I	A	Q	N
KJ813836/Fisher/ND/75/2013/USA		L	T	D	D	S	F	Y	T	Q	N
KT162038/BJ14–28/2014/China	New CPV-2a	L	T	G	Y	A	Y	I	A	Q	N
DQ340428/BR209–94/1994/Brazil		L	T	G	Y	A	F	Y	T	Q	N
EU009200/K001/2007/South Korea		L	T	G	Y	A	F	Y	A	Q	N
DQ340409/BR183–85/1985/USA	CPV-2b	L	T	G	Y	S	F	Y	T	Q	D
FJ005261/G82/1997/Germany		L	T	G	Y	S	F	Y	T	Q	D
JQ743891/CPV-10/2010/China		L	T	G	Y	A	Y	I	A	Q	D
GQ857608/CPV07–06/2007/China	New CPV-2b	L	T	G	Y	A	Y	Y	T	Q	D
AB054222/LCPV V139/2001/Japan		L	T	G	Y	A	F	Y	A	Q	D
JQ743890/CPV-4/2011/China		L	T	G	Y	A	Y	I	T	Q	D
FJ005195/136/2000/Italy	CPV-2c	L	T	G	Y	A	F	Y	T	Q	E
FJ005201/G362/1997/Germany		L	T	G	Y	A	F	Y	T	Q	E
KP260509/BJ14–9/2014/China		L	T	G	Y	A	Y	I	T	R	E
KU244254/NPUST014/2015/Taiwan		L	T	G	Y	A	Y	I	G	R	E

### Composition of the CPV VP2 gene

The nucleotide content of the analyzed sequences were calculated (Additional file 1: Table S2). The A%, T%, C%, and G% were 34.98 ± 0.118, 29.15 ± 0.66, 15.79 ± 0.113, and 19.8 ± 0.093 (mean ± standard deviation; mean ± SD) respectively. This indicates that all the selected sequences are A/T rich with subtle differences among the CPV VP2 sequences. Additionally, the codon composition at the third position including A3, G3, T3, C3, and GC3 were calculated with mean ± SD of 56.69 ± 0.4086, 8.97 ± 0.3638, 53.68 ± 0.3296, 5.91 ± 0.3346, and 32.62 ± 0.4391 further suggesting that A/T terminated codons might be more abundant than G/C terminated codons. Accordingly, the GC composition of CPV VP2 was low, ranging from 34.9% to 36.75% (mean 35.67%, SD ± 0.1672) compared with other vertebrate DNA viruses.

### Synonymous codon usage bias among CPV VP2 genes

The various RSCU of synonymous codons of the CPV VP2 gene were calculated to decrypt the degree of preferred A/T-terminated codons (Table 2). Among the eighteen most abundant codons, all were A/T terminated codons including ten T-terminated codons (TGT for Cys, GAT for Asp, TTT for Phe, GGT for Gly, CAT for His, ATT for IIe, AAT for Asn, TAT for Tyr, GTT for Val, and TCT for Ser) and eight A-terminated codons (GCA for Ala, GAA for Glu, AAA for Lys, TTA for Leu, CCA for Pro, CAA for Gln, AGA for Arg, and ACA for Thr). This indicates codon usage bias in synonymous codons. This together with the nucleotide composition analysis indicates that the usage of optional codons might be influenced by compositional constraints resulting in the presence of mutational pressure.

**Table 2:** RSCU analysis of the 59 synonymous codons of CPV VP2 gene

AA	Codon	CPV	AA	Codon	CPV
A(Ala)	GCA	***2.271***	P(Pro)	CCA	***2.972***
	GCC	0.113		CCC	0.007
	GCG	0.325		CCG	0.006
	GCT	1.292		CCT	1.015
C(Cys)	TGC	0.059	Q(Gln)	CAA	***1.824***
	TGT	***1.941***		CAG	0.176
D(Asp)	GAC	0.138	R(Arg)	AGA	***5.451***
	GAT	***1.863***		AGG	0.019
E(Glu)	GAA	***1.586***		CGA	0.009
	GAG	0.414		CGC	0.003
F(Phe)	TTC	0.074		CGG	0.259
	TTT	***1.926***		CGT	0.259
G(Gly)	GGA	1.483	S(Ser)	AGC	0.219
	GGC	0.225		AGT	1.774
	GGG	0.524		TCA	1.204
	GGT	***1.767***		TCC	0.002
H(His)	CAC	0.215		TCG	0.001
	CAT	***1.785***		TCT	***2.801***
I(IIe)	ATA	0.710	T(Thr)	ACA	***1.925***
	ATC	0.130		ACC	0.176
	ATT	***2.160***		ACG	0.141
K(Lys)	AAA	***1.889***		ACT	1.758
	AAG	0.111	V(Val)	GTA	1.569
L(Leu)	CTA	1.201		GTC	0.112
	CTC	0.001		GTG	0.431
	CTG	0.005		GTT	***1.889***
	CTT	0.518	Y(Tyr)	TAC	0.204
	TTA	***2.747***		TAT	***1.796***
	TTG	1.529			
*N(Asn)*	*AAC*	0.488			
	*AAT*	***1.512***			

The eighteen abundant codons are represented in bold and Italic

Moreover, the effective number of codons (ENC) values were calculated to quantify the extent of codon usage bias of the CPV VP2 gene. ENC values ranged from 31.85 to 34.83 with an average value of 32.62 and a SD of 0.43 indicating a high codon usage bias.

### Mutational pressure shaping codon usage bias

**Fig. 2:**
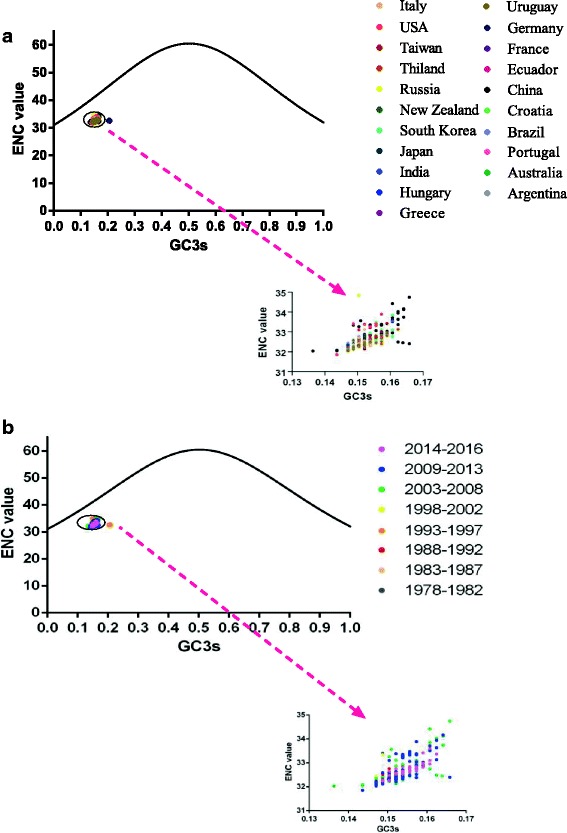
ENC plots displaying the relationships between ENC and the GC content at the third codon position (GC3s) in relation to geographical distribution (**a**) or time of isolation (**b**) depicted in different colors

ENC plots were constructed according to the geographical distribution and isolation time (Fig. 2a and b respectively). The ENC plots show that not all the different sequences are positioned on the standard curve, indicating that mutational pressure is not the sole factor shaping codon usage bias but other forces, including geographical distribution, could play a role too. Additionally, sequences isolated from different countries and years clustered together with slight fluctuation except for sequences isolated from Germany in 1995. This result is consistent with the small SD of the ENC.

To explain the influence of mutational pressure on synonymous codon usage bias in-depth, correlation analyses was performed between the nucleotide compositions (A%, T%, G%, C%, and GC%) and codon compositions (A3s, T3s, G3s, C3s, and GC3s) and the ENC values (Additional file 1: Table S3). The nucleotide contents correlated with composition (*p* < 0.01), excluding the relationship between T% and the A3 and C3, and GC% with the A3, G3. These results indicate that nucleotide composition constrains synonymous codon usage bias and hence mutational pressure.

**Fig. 3:**
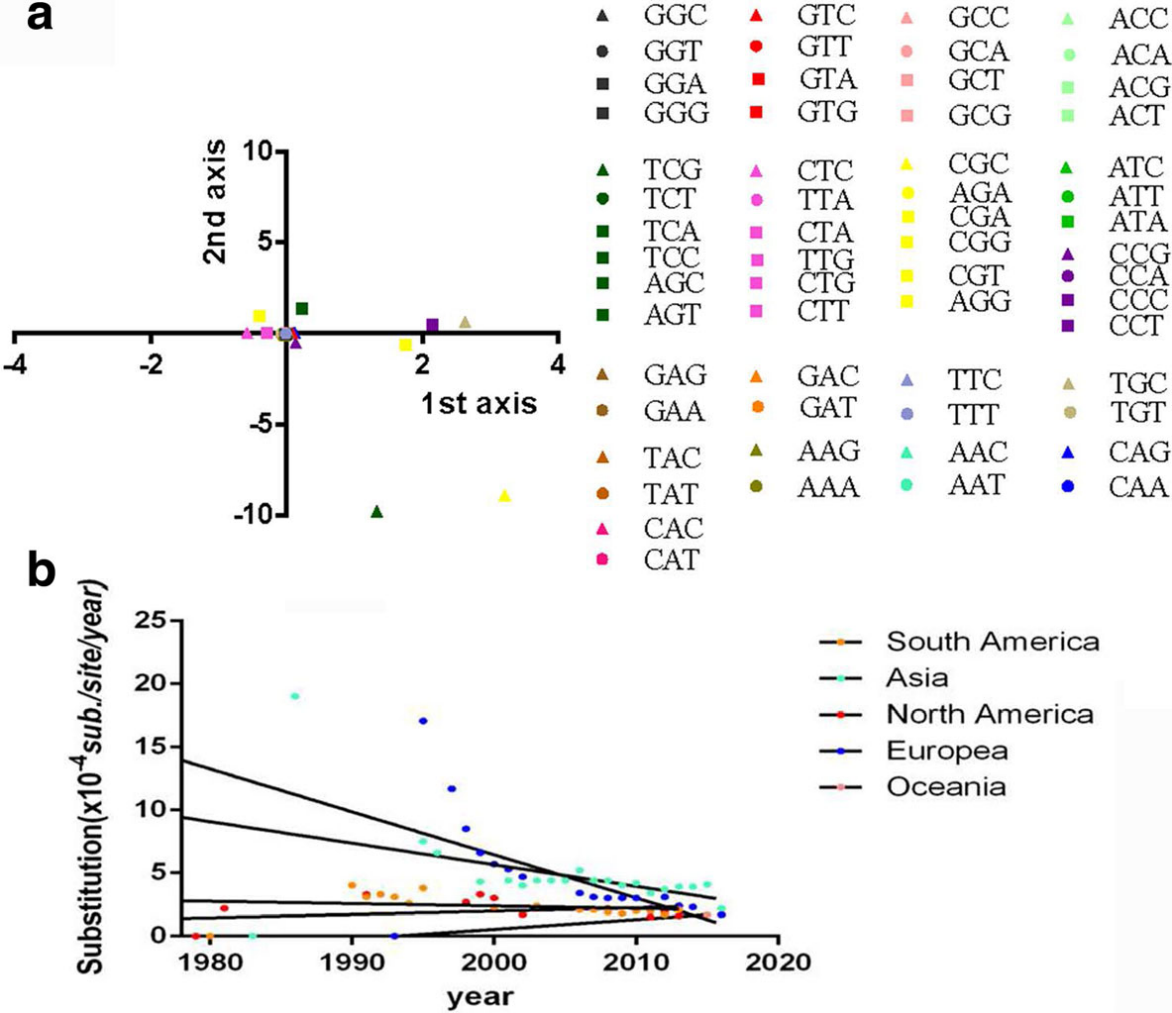
**a** CoA analysis based on RSCU of 424 CPV VP2 genes. The most frequent codon is represented by *circles* and the rarest codon is represented by *triangles*. The remaining codons are represented by *squares*. **b** Evolutionary rate of the CPV VP2 gene. Each evolution rate represents the substitution numbers of each sequence compared with the earliest sequence (sub./site/year). The South American clades are represented in *orange*, the Asian clades in *light blue*, the North American clades in *red*, the European clades in *dark blue*, and the Oceania clades in *pink*

To analyze the variation of codon usage in the CPV VP2 gene, CoA analysis—a multivariable method—was employed [22] (Fig. 3a). The analysis suggested that the first four principal axes accounted for 60.56% of the total variation with the first, second, third, and fourth principal axis accounting for 23.17%, 14.65%, 13.2%, and 9.55%, respectively, suggesting that the variation of RSCU of synonymous codons were contributed by the first and second axis, this phenomenon regarded as the tendency of codon usage bias. To understand the distribution of synonymous codons, the first and second axes values were plotted against each other. The CoA-RSCU analysis revealed that all of the eighteen frequently employed codons—ending with A/T—clustered near the origin with slightly deviation from each other. Additionally, the remarkable correlation between the first axis and codon compositions demonstrated that nucleotide composition contributed to codon usage.

### Evolutionary rate analysis

The evolutionary rate of the CPV2 gene in the five continents was estimated (Fig. 3b) revealing that the substitution rates of strains isolated from Asia and Europe descend over time (−1.17 × 10^−5^ and −3.4 × 10^−5^ respectively). However, sequences collected from Oceania, South America, and North America increased with rates of 7.8 × 10^−6^, 1.9 × 10^−6^, and 2.6 × 10^−6^ respectively. Additionally, the Asian and European sequences experienced wider substitution ranges, not falling within the regression lines.

### The role of natural selection in shaping codon usage bias

To determine the role of natural selection in shaping codon usage of CPV VP2, the correlations between codon usage composition (A3s, T3s, G3s, C3s, GC3s) and Gravy and Aroma were calculated (Additional file 1: Table S3). The result shows that both Gravy and Aroma were significantly correlated with codon usage composition (*p* < 0.01) while Gravy correlated with T3 s with a higher *p* value (0.01 < *p* < 0.05), revealing that translational selection is one factor affecting codon usage during the evolution of CPV.

### The role of dinucleotide abundance driving the codon usage of CPV VP2

Analysis of the sixteen dinucleotides (Additional file 1: Table S4) showed that no dinucleotide frequency equaled to the expected value, thus no dinucleotide was randomly used. Over-represented dinucleotides were TpG, GpG, CpA, while under-represented dinucleotides were CpG and TpC. The most under-represented dinucleotide was CpG, in accordance with the fact of GC being the lowest nucleotide composition and that none of the eighteen most frequently used synonymous codons ended in G/C. This indicates that the dinucleotide frequency shapes the codon usage of the CPV VP2 gene.

### The decisive factor in shaping codon usage bias—Mutational pressure vs. natural selection

**Fig. 4:**
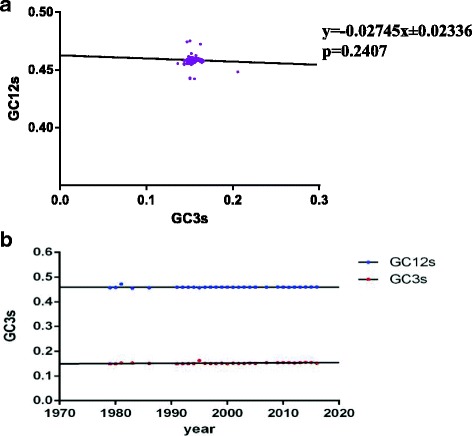
**a** Neutrality analysis displaying GC3s plotted against GC12s.The line regression was −0.2745× ± 0.2336, and GC3s was not correlated with GC12s, with *p* > 0.1. **b** Relationship of GC3s or GC12s and year of isolation. GC3s is represented in *red*; GC12s is represented in *blue*

Based on the analysis above, it can be concluded that the codon usage pattern was influenced not only by mutational pressure but also by natural selection. Therefore, to determine the decisive one shaping codon usage bias, neutrality analysis was performed (Fig. 4a). The neutrality analysis showed that the GC12 value did not correlate with the GC3 value (R^2^ = 0.003261). The slope of the regression line was −0.02745 ± 0.02336, with *p* > 0.01, indicating no correlation between GC3s and GC12s. Thus, natural selection dominates directional mutation pressure in driving the codon usage pattern.

Moreover, the evolutionary rate was plotted by GC12s or GC3s against evolution time (Fig. 4b). The plot shows that GC12 is negatively correlated with year of isolation, while the GC3 is positively correlated with year of isolation, suggesting that the role of mutational pressure is increasing with CPV evolution.

### Other factors shaping codon usage bias

**Fig. 5:**
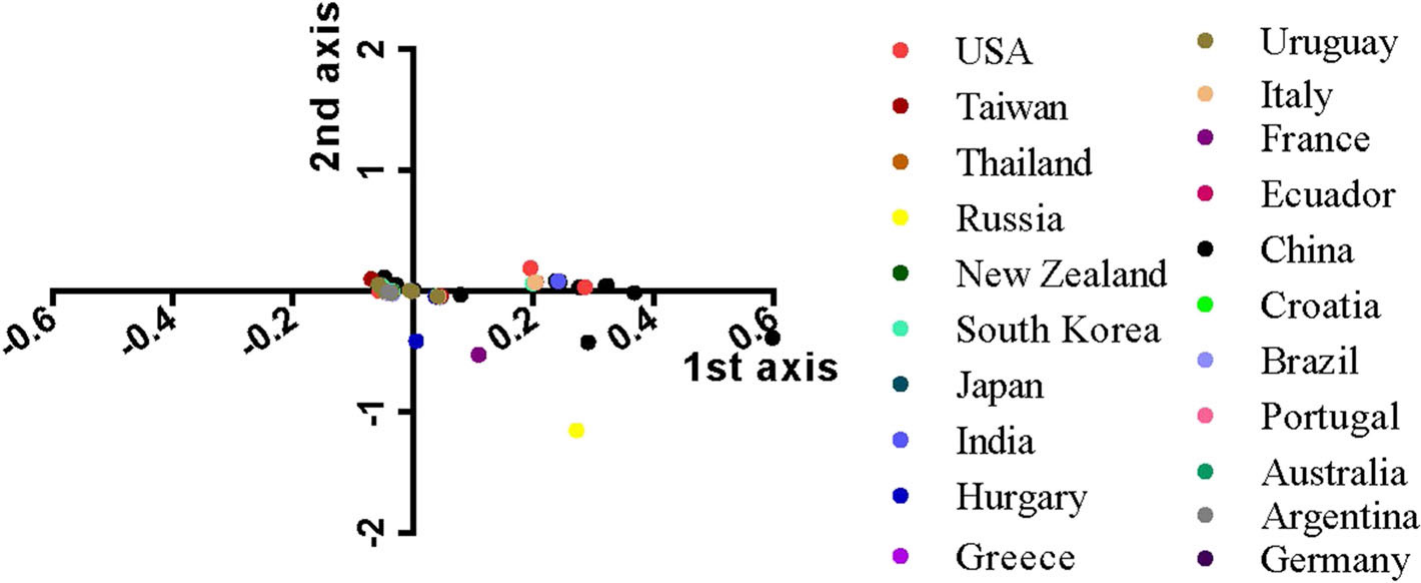
CoA analysis against geographical distribution. Different geographical distributions are represented by different colors

Previous studies have shown that geographical distribution also contributes to codon usage patterns [22]. To investigate if this is the case for the CPV VP2 gene, the CoA according to isolated areas were analyzed (Fig. 5). We found that different sequences isolated from different countries diverged from each other, in particular sequences isolated from China, France, and Russia. Notably, isolates from the same country did not cluster together, such that the Chinese sequences exhibited a significant distribution, indicating that the CPV VP2 gene experienced mutation during its evolution.

## Discussion

Since the emergence of canine parvovirus, there has been a lack of systematic genomic analysis on the evolution of CPV type 2 VP2 gene. This study represents a large-scale comprehensive analysis, as phylogenetic analysis and mutant analysis combined with codon usage analysis was performed.

CPV VP2 is a main structural protein, which determines the major mutations during the evolution of CPV. CPV has some persistent genetic variations and at present CPV-2a, CVP-2b, and CPV-2c are the three major antigenic variants of origin CPV type 2 [13, 23]. The phylogenetic analysis performed here showed that CPV VP2 diverged into 6 clades: GI, GII, GIII, GIV, GV, GVI and there are no differences in the geographical distribution of the sequences.

The phylogeny revealed that the GI, GII, and GIII group mainly include the early CPV-2a sequences. The genotype of the CPV-2b antigenic variant mainly belongs to the GVIc group worldwide. Almost all the CPV-2c strains formed another independent subgroup, named group GVId. It is important to note that the CPV-2a, CPV-2b, and CPV-2c variants do not cluster into to the same clades based on the phylogeny. This is because the classification into genotypes is based on mutation sites while the phylogeny is reconstructed based on nucleotide relationships. Nowadays, CPV-2a and CPV-2b are predominant in Asian countries including Korea, China, Thailand, Japan, Taiwan, and India. Outside of Asia, CPV-2a and 2b isolates are common in the United States, whereas CPV-2c is more widespread in Uruguay, Brazil, and Argentina [24]. The phylogenetic analysis demonstrated the evolution of CPV VP2 from a macro-perspective, while the detailed changes were analyzed by amino acid mutant analysis and codon usage analysis. It is considered that CPV, as the original type 2 (origin CPV type 2), emerged in 1978 and is a specific variant of feline panleukopenia virus [25]. Several amino acid mutations (M87 L, I101T, A300G, D305Y) in CPV 2a [26] as well as the N426D mutation in VP2 of CPV-2b [27] result in biological differences between CPV-2 and CPV-2a, CPV 2b, respectively, including antigenic reactivity to monoclonal antibodies, binding affinity to the feline transferrin receptor, and the ability to replicate in cats [27–29]. In addition, a third variant carrying a N426E mutation, that is critical to distinguish CPV-2a, CPV-2b and CPV-2c strains (Table 1), was discovered in Italy in 2000 [12].

New variants discovered in CPV-2a and CPV-2b carrying substitutions on site 297 were previously identified and designated as new CPV-2a and new CPV-2b, respectively [30, 31]. This site is under strong positive selection and may have had a remarkable influence on the process of host adaptation [32]. This site is defined as a marker in identifying the new CPV-2a and new CPV-2b variants. Except for these mutations, a Q370R mutation was only found in some CPV-2c strains isolated from China and Taiwan. Previous studies have suggested that this is evidence of a potential CPV-2c variant or new CPV-2c. Further studies regarding the potential variant CPV-2c strains should be conducted to understand the relationship between the Q370R substitution and viral pathogenicity [33].

In addition, we found other mutations (F267Y, Y324I and T440A) in genotype new CPV 2a and 2b, which had been previously described. These may contribute to virus immune escape via antigenic drift and consequent vaccine failure [33–36]. Among the non-synonymous mutations, amino acid residue 267 is not exposed on the capsid surface and thus substitutions in this position may not affect the antigenicity of the virus [37]. The T324I mutation in CPV-2a strains may be a common amino acid alteration in Asian countries, especially in China [33] and in South America. Mutations at amino acid residue 324 may have an impact on parvovirus host range [38] and it was also been demonstrated that the T324I mutation occurs in regions referring to potentially important antigenic epitopes. This substitution might have a direct influence on viral biology [5]. The T440A mutation is also important for CPV because residue 440 is located on the top of the three-fold spike of the VP2 protein on the surface of the capsid, the main viral antigenic site [39, 40]. Therefore, resulting in the emergence of further antigenic variants. Overall, further studies on the effects of the F267Y, Y324I, and T440A mutations on CPV VP2 are imperative. Additionally, we found amino acid substitutions: K80R, K93 N, V103A and D323N, in agreement with previous studies [27, 41, 42].

Our study has several limitations. Firstly, not all CPV sequences were included. For example, sequences collected from Oceania are not complete and thus not included, which might impair the correct characterization of the geographical distribution of CPV in New Zealand in comparison with the study of Ohneiser et al. [43]. Secondly, the sequences analyzed are very similar to each other with 99.7% identity. Since the sequences were obtained from GenBank and probably obtained by PCR amplification, we have no information on the levels of fidelity of the enzymes that were used and measures to control for PCR bias.

The evolutionary rate of the CPV VP2 gene was estimated for the first time according to the geographical distribution in different continents, which revealed that the evolutionary rate of CPV VP2 in Asia and Europe decreased.

Furthermore, to understand the molecular evolution of CPV from 1979 to 2016, the codon usage of the 424 coding sequences of the CPV type 2 VP2 gene were analyzed. Previous studies demonstrated that the degree of codon usage bias is different among the different species, even for genes belonging to the same species [44–47]. It is considered that mutation pressure [48, 49] or natural selection [50, 51] are the two major factors affecting codon usage bias, as well as other factors such as secondary mRNA structure [52], tRNA abundance [53], geographical distribution [54], and external environment [55]. To date, most studies on codon usage bias focus on RNA viruses and their complete genomes. There are few studies on DNA viruses and in single genes. Here, we firstly analyzed the nucleotide composition of CPV VP2 and we found that the A/T content was higher than the G/C content and that A/T terminated codons were preferred than G/C terminated codons. Furthermore, most of the eighteen most frequently used codons ended in A/T. Altogether this indicates that there is codon usage bias in the CPV VP2 gene.

Here ENC analysis was employed to evaluate the codon usage bias of the VP2 gene of CPV. Compared with previously reported DNA viruses such as porcine circovirus [56] (mean value 56.80), human herpesvirus [57] (range from 39.47 ± 4.81 to 54.92 ± 3.26), duck plague virus UL35 gene [58] (mean value 47.55), duck enteritis virus UL24 gene [59] (range from 40.10 to 60.44), and iridovirus [60] (range from 35.87 to 51.81), canine parvovirus [61] exhibits high codon usage bias (36.46), in accordance with the study of Shackelton et al. [20]. Interestingly, we found that among the above-mentioned DNA viruses, porcine circovirus exhibits lower codon usage while CPV exhibits much higher codon usage bias. This might be the result of small DNA viruses replicating in cells that are mitotically active [20] and the CPV having a strict host range, for example, the origin CPV type 2 is only infectious in dogs, while CPV 2a and CPV 2b infect both dogs and felines.

In this study, the ENC-plot analysis showed that all points representing different sequences were lower than the theoretical curve, which is suggestive of mutation pressure contributing to the codon usage pattern and also revealing that other factors also influence the codon usage of the CPV VP2 gene. Mutation pressure and natural selection were proven to be the two main factors shaping codon usage bias. To explore the role of mutation pressure, the correlation between nucleotide content and codon composition was analyzed and showed a strong correlation. However, the relationship between T%, A3s and C3 indicated that the codon usage pattern was influenced by mutation pressure. Further, the CoA based on RSCU analysis demonstrated the role of mutation pressure. Thus, mutation pressure is essential in shaping the codon usage of the VP2 gene of CPV in accordance with previous studies showing that mutational bias is important in shaping the codon usage pattern of DNA viruses [20].

To demonstrate the possible role of natural selection, a strong correlation between nucleotide content and Gravy/Aroma was revealed, suggesting that natural selection is more important shaping the codon usage than mutation pressure. This was significantly demonstrated by neutrality analysis in which the natural selection constrains the codon usage bias (97.25% coverage). In conclusion, high codon usage bias was observed for the VP2 gene of CPV, which was mainly caused by natural selection, in contrast to porcine circovirus that is mainly driven by mutation pressure [62]. In addition, the results reveal that the suppression of CpG in CPV VP2, which might due to the innate immune system of host treatment of unmethylated CpGs, is a pathogen characteristic. Also, geographical distribution is correlated with the codon usage pattern of VP2 by CoA analysis based on the country of isolation. Thus, codon usage analysis, natural selection, mutation pressure, dinucleotide abundance and geographical distribution are essential.

## Conclusions

In conclusion, a total of 424 sequences of the CPV type 2 were analyzed and new viewpoints regarding phylogenetic relationships, amino acid mutations, and codon usage were discovered including: CPV VP2 sequences can be classified into six clades GI, GII, GIII, GIV, GV, and GVI; the origin CPV type 2 sequences mainly belong to Group GI and GII; genotype CPV 2a cluster into clades GIII, GVd, GVIa and GVIb; CPV 2b sequences cluster into clades GVd, and GVIc; and CPV-2c cluster into group GVId. Additionally, the substitution rates of sequences isolated from Asia and Europe descended over time. Most importantly, natural selection is the force that has the biggest impact in driving the codon usage of CPV. These new results regarding CPV evolution will greatly serve future CPV research.

## Materials and methods

### Sequence data

A total of 424 reference sequences were extracted from the National Center for Biotechnological Information (NCBI) website (https://www.ncbi.nlm.nih.gov/) including 744,120 codons. The complete coding sequences (CDS) of CPV type 2 VP2 were 1755 bp in length, representing 21 countries across the world and isolated from 1979 to 2016. Detailed information is listed in Additional file 1: Table S5.

### Phylogenetic and amino acid analysis

Maximum clade credibility (MCC) trees were inferred using Bayesian evolutionary analysis by sampling trees (BEAST, version 1.8.4, http://beast.bio.ed.ac.uk) using HKY as the nucleotide substitution model with gamma-distributed rate heterogeneity and a relaxed molecular clock. The Markov chain Monte Carlo (MCMC) algorithm was run for 100-million generations; 10 % of the chain was removed as burn-in. The time to the most recent common ancestor (tMRCA) was estimated. It is essential to note that the value of the Bayesian posterior probabilities indicate the support value of each node. Bayesian posterior probability >0.5 of the relative clades were displayed in the tree. The posterior probability of clades GVId was 0.23, while the GVI clade with value less than 0.1, due to the similarity of the relative sequences. Amino acid mutations and mutation sites were analyzed using the DNASTAR software.

### Evolutionary rate of the CPV VP2 gene

The evolutionary rate was calculated as the number of substitutions per site per year. The evolutionary rate of the sequences derived from each continent was analyzed and compared to the earliest identified sequence for that continent, respectively. The substituted sites were aligned by method of Align by ClustalW using the MEGA7.0 software. Linear regression was calculated using Graph Pad Prism 6.0.

### Codon usage measurement indices

#### Nucleotide biases

The frequencies of the related coding sequences for nucleotide content (A%, C%, T%, and G%) were analyzed using BioEdit. The synonymous codons on the third position (A3%, C3%, T3%, and G3%) were calculated using the CodonW (http://mobyle.pasteur.fr/cgi-bin/portal.py?#forms::CodonW) without confusing the amino acid composition or the overall G + C at the third codon positions. In addition, cusp (http://mobyle.pasteur.fr/cgi-bin/portal.py?#forms::cusp) was employed to measure the G + C at the first and second positions of the synonymous codons.

#### The effective codon usage statistic-ENC

The magnitude of codon usage of relative viruses were reflected by the ENC [63]. ENC values range from 20 to 61, with a score of 20 signifying severe bias (only one codon being employed in each synonymous codon) and a score of 61 denoting no bias (the frequency of all employed codons are equal in coding amino acids). The ENC was calculated as the given formula:

$$ \mathrm{ENC}=2+\frac{9}{{\overline{\mathrm{F}}}_2}+\frac{1}{{\overline{\mathrm{F}}}_3}+\frac{5}{{\overline{\mathrm{F}}}_4}+\frac{3}{{\overline{\mathrm{F}}}_6} $$

In the i-fold degenerate amino acids, F (*i* = 2,3,4,6) represents Fi values of them. An ENC value less than 35 is considered a stronger indication of codon usage bias [64].

#### ENC-plot analysis

The ENC-plot is an absolute statistic to evaluate the decisive factor in shaping codon usage bias. ENC values were taken into account for the background of the GC3s [63]. If codon usage is constrained only by G + C mutation bias, the expected ENC values would simply lie on or around the standard curve. Alternatively, other factors such as natural selection may play a major role in shaping codon usage bias. The ENC plots were calculated using the following formula:

$$ \mathrm{ENC}-\mathrm{plot}=2+\mathrm{s}+\left(\frac{29}{s^2+{\left(1+ s\right)}^2}\right) $$

where s is the GC content at the third codon position, namely the GC3s.

#### Relative synonymous codon usage analysis

Relative synonymous codon usage (RSCU) was previously employed to estimate the codon usage bias of a particular gene or genome [65], using the ratio of observed frequency to theoretical frequency [66], with the formula given below:

$$ \mathrm{RSCU}=\frac{g_{i j}}{\sum_j^{n_i}{g}_{i j}}{n}_i $$

It’s essential to note that *g*_*ij*_ represent the *i*_*th*_ codon of the *j*_*th*_ amino acid, and *n*_*i*_ represent the kinds of synonymous codons [67]. All codons, except Met, Trp, and termination codons were used equally for the corresponding amino acid. Without the influence of the amino acid frequency, the RSCU matrix was considered the preferred method. In contrast to the ENC, larger RSCU values indicate stronger codon usage bias, such that RSCU values equal to 1, means no bias, more than 1 indicates positive codon usage bias, otherwise, negative codon usage bias.

#### General average hydrophobicity (Gravy) and aromaticity (Aroma) analysis

The Gravy and Aroma scores represent a particular amino acid usage, which result from translation selection, namely natural selection. Both the Gravy and Aroma scores were calculated using CondoW (http://mobyle.pasteur.fr/cgi-bin/portal.py?#forms::CodonW) [68]. The hydrophobicity of each amino acid is denoted by the Gravy index [26]. Similarly, the occurrence of aromatic amino acids (Phe, Trp and Tyr) is revealed by the Aroma value.

#### Multifactor variable-CoA analysis

To analyze the variation of codon usage data, CoA analysis—a multivariable method—was employed [22]. The RSCU of all the selected sequences was used to measure the synonymous codon usage pattern. The RSCU values of all codons were distributed into a 59-dimension space (excluding for three terminated codons, Met and Trp) and transformed into unrelated factors. Therefore, the factors affecting codon usage bias were detected [69, 70]. In this study, excluding the influence of unequal usage of amino acid, the axes were reduced to 40, even though the synonymous codons were 59 [71]. In this analysis, CoA was plotted against RSCU. Moreover, CoA based on the isolated locations was analyzed.

#### Neutral evolution analysis

To differentiate the varying role of mutation pressure and natural selection, neutral analysis was employed [72]. Neutrality plots represent the GC12s against the GC3s. Each independent dot represents a selected sequence. The regression line was employed to explain the major role of mutation pressure as opposed to natural selection. In addition, the evolutionary rate was explained by GC12s or GC3s plotted against an isolated year which was drawn using Graph Pad Prism 6.0.

#### Dinucleotide frequency analysis

Dinucleotide abundance correlates with codon usage bias [73], therefore, the sixteen dinucleotide compositions of the coding sequences of VP2 representing 424 sequences were calculated using the software DAMBE using the following formula as previously described [74]:

$$ {P}_{x y}=\frac{f_{x y}}{f_y{f}_x} $$

*f*_*x*_ , *f*_*y*_ represent the occurance of the nucleotide X, Y respectively, while *f*_*xy*_ denotes the observed occurrence of dinucleotide XY. *f*_*x*_ *f*_*y*_ denote the expected occurance of dinucleotide XY. It is considered that *P*_*xy*_ > 1.23 suggests over-represented while *P*_*xy*_ <0.78 suggests under-represented.

### Correlation analysis

Correlation analysis of nucleotide composition with A3s, T3s, G3s, C3s, GC3s, Aroma, Gravy, ENC, 1st axis and 2nd axis were calculated using Graph Pad Prism 6.0.

## Additional file

Additional file 1:
**Table S1.** The detail numbers of the three discovered mutation sites of the 424 sequences. **Table S2.** The nucleotide contents of the selected sequences and the mean ± SD values of the A%,T%,G%,C%,respectively. **Table S3.** The correlation analysis of codon usage indices. *Signifies 0.05 > *p* > 0.01; **signifies *p* < 0.01. **Table S4.** The abundance of the 16 dinucleotides. **Table S5.** The detail information of the 424 sequences. (DOCX 96 kb)

## Abbreviations

COA: Correspondence analysis
CPV-2: Canine parvovirus type 2
ENC: Effective number of codons
PCA: Principal component analysis
RSCU: The relative synonymous codon usage
