# Supplementary material for: Evolutionary and genetic analysis of the VP2 gene of canine parvovirus
Source: BMC Genomics. 2017 Jul 17;18:534. doi: 10.1186/s12864-017-3935-8 (PMC5512735; doi:10.1186/s12864-017-3935-8)
Supplement: Additional file 1: — Table S1. The detail numbers of the three discovered mutation sites of the 424 sequences. Table S2. The nucleotide contents of the selected sequences and the mean ± SD values of the A%,T%,G%,C%,respectively. Table S3. The correlation analysis of codon usage indices. *Signifies 0.05 > p > 0.01; **signifies p < 0.01. Table S4. The abundance of the 16 dinucleotides. Table S5. The detail information of the 424 sequences. (DOCX 96 kb) [file 12864_2017_3935_MOESM1_ESM.docx]

**Table S1 The detail numbers of the three discovered mutation sites of the 424 sequences.**

| **Amino acids** | | **Number** |  |  |
| --- | --- | --- | --- | --- |
| **267-F** | 11 | 41 | 196 | 93 |
| **267-Y** | 0 | 0 | 27 | 55 |
| **324-Y** | 11 | 41 | 171 | 80 |
| **324-I** | 0 | 0 | 53 | 68 |
| **440-T** | 11 | 41 | 185 | 96 |
| **440-A** | 0 | 0 | 39 | 47 |
| **Year** | 1979-1990 | 1991-2000 | 2001-2010 | 2011-2016 |

**Table S2 The nucleotide contents of the selected sequences and the mean±SD values of the A﹪,T﹪,G﹪,C﹪,respectively.**

| ***Number*** | ***A%*** | ***C%*** | ***G%*** | ***T%*** |  | ***Number*** | ***A%*** | ***C%*** | ***G%*** | ***T%*** |  |
| --- | --- | --- | --- | --- | --- | --- | --- | --- | --- | --- | --- |
| KM236573 | 35.27 | 15.78 | 19.66 | 29.29 |  | GQ865519 | 35.16 | 15.73 | 19.83 | 29.29 |  |
| KM236570 | 35.27 | 15.90 | 19.72 | 29.12 |  | GQ865518 | 35.16 | 15.73 | 19.83 | 29.29 |  |
| KM236569 | 35.16 | 15.84 | 19.83 | 29.17 |  | KF539789 | 35.04 | 15.90 | 19.83 | 29.23 |  |
| KM236568 | 35.16 | 15.78 | 19.83 | 29.23 |  | KF539790 | 34.99 | 15.84 | 19.89 | 29.29 |  |
| JF414822 | 35.10 | 15.78 | 19.89 | 29.23 |  | KF539791 | 35.10 | 15.95 | 19.77 | 29.17 |  |
| JF414825 | 35.04 | 15.84 | 19.89 | 29.23 |  | KF539792 | 35.04 | 15.78 | 19.83 | 29.34 |  |
| JF414826 | 35.27 | 15.84 | 19.77 | 29.12 |  | KF539797 | 35.10 | 15.67 | 19.83 | 29.40 |  |
| JF414823 | 35.10 | 15.78 | 19.89 | 29.23 |  | KF539802 | 35.04 | 15.78 | 19.77 | 29.40 |  |
| JF414820 | 35.10 | 15.78 | 19.89 | 29.23 |  | KF539803 | 35.21 | 15.67 | 19.66 | 29.46 |  |
| JF414821 | 35.10 | 15.78 | 19.89 | 29.23 |  | KF539804 | 35.10 | 15.73 | 19.77 | 29.40 |  |
| JF414818 | 35.10 | 15.78 | 19.89 | 29.23 |  | KF539805 | 34.99 | 15.84 | 19.83 | 29.34 |  |
| JF414819 | 35.04 | 15.73 | 19.94 | 29.29 |  | AB120720 | 35.16 | 15.78 | 19.72 | 29.34 |  |
| JF346754 | 35.33 | 15.73 | 19.66 | 29.29 |  | AB120723 | 35.10 | 15.73 | 19.77 | 29.40 |  |
| JF414817 | 35.27 | 15.84 | 19.72 | 29.17 |  | AB120724 | 35.16 | 15.84 | 19.77 | 29.23 |  |
| KU508691 | 35.16 | 15.73 | 19.83 | 29.29 |  | AB120728 | 35.10 | 15.78 | 19.77 | 29.34 |  |
| KU508692 | 35.16 | 15.73 | 19.83 | 29.29 |  | AB128923 | 35.04 | 15.73 | 19.83 | 29.40 |  |
| KU508693 | 35.16 | 15.73 | 19.89 | 29.23 |  | AB115504 | 35.10 | 15.67 | 19.83 | 29.40 |  |
| KT275255 | 35.21 | 15.78 | 19.83 | 29.17 |  | AB054214 | 35.16 | 15.73 | 19.77 | 29.34 |  |
| KT275256 | 35.16 | 15.73 | 19.83 | 29.29 |  | AB054220 | 34.99 | 15.78 | 19.89 | 29.34 |  |
| KR559896 | 34.99 | 15.95 | 19.89 | 29.17 |  | AB054221 | 35.04 | 15.78 | 19.83 | 29.34 |  |
| KT275254 | 35.04 | 15.78 | 19.94 | 29.23 |  | AB054222 | 35.10 | 15.78 | 19.77 | 29.34 |  |
| KR559895 | 35.04 | 15.84 | 19.94 | 29.17 |  | AB054223 | 35.10 | 15.73 | 19.77 | 29.34 |  |
| KT275252 | 35.10 | 15.78 | 19.89 | 29.23 |  | AB054224 | 35.10 | 15.84 | 19.77 | 29.29 |  |
| KT275253 | 35.10 | 15.78 | 19.83 | 29.29 |  | D78585 | 34.99 | 15.78 | 19.89 | 29.34 |  |
| KR559891 | 35.04 | 15.84 | 19.83 | 29.29 |  | KP893077 | 34.93 | 15.95 | 20.00 | 29.12 |  |
| KR559892 | 35.10 | 15.84 | 19.89 | 29.17 |  | KP893078 | 35.04 | 15.90 | 19.89 | 29.17 |  |
| KR559893 | 35.16 | 15.78 | 19.83 | 29.23 |  | EF599098 | 34.99 | 15.90 | 19.89 | 29.23 |  |
| KR559894 | 35.16 | 15.73 | 19.83 | 29.29 |  | EU009206 | 34.87 | 16.01 | 19.89 | 20.23 |  |
| DQ340433 | 35.10 | 15.73 | 19.77 | 29.40 |  | EU009205 | 34.73 | 15.90 | 19.89 | 29.29 |  |
| DQ340434 | 35.10 | 15.73 | 19.77 | 29.40 |  | EU009200 | 35.04 | 16.01 | 19.77 | 29.17 |  |
| DQ340430 | 35.16 | 15.84 | 19.66 | 29.34 |  | EF599096 | 35.10 | 15.78 | 19.77 | 29.34 |  |
| DQ340432 | 35.10 | 15.90 | 19.77 | 29.23 |  | FJ197823 | 34.93 | 15.73 | 19.94 | 29.40 |  |
| DQ340425 | 35.10 | 15.73 | 19.77 | 29.40 |  | FJ197830 | 34.93 | 15.84 | 20.00 | 29.23 |  |
| DQ340426 | 35.10 | 15.73 | 19.77 | 29.40 |  | FJ197831 | 34.87 | 15.95 | 20.00 | 29.17 |  |
| DQ340428 | 35.04 | 15.73 | 19.83 | 29.40 |  | FJ197832 | 35.10 | 16.01 | 19.77 | 29.12 |  |
| DQ340429 | 35.04 | 15.73 | 19.83 | 29.40 |  | FJ197839 | 34.93 | 15.73 | 19.83 | 29.52 |  |
| DQ340422 | 35.04 | 15.73 | 19.83 | 29.40 |  | FJ197840 | 34.87 | 15.84 | 20.00 | 29.29 |  |
| DQ340419 | 35.16 | 15.73 | 19.77 | 29.34 |  | FJ197841 | 34.93 | 15.90 | 19.89 | 29.29 |  |
| DQ340420 | 35.10 | 15.73 | 19.77 | 29.40 |  | FJ197842 | 34.81 | 15.90 | 20.06 | 29.23 |  |
| DQ340421 | 35.04 | 15.73 | 19.83 | 29.40 |  | FJ197845 | 34.81 | 15.84 | 20.00 | 29.34 |  |
| DQ340415 | 35.10 | 15.73 | 19.77 | 29.40 |  | EF189717 | 35.04 | 16.07 | 19.83 | 29.06 |  |
| DQ340417 | 35.10 | 15.73 | 19.77 | 29.40 |  | AY742933 | 35.10 | 15.78 | 19.77 | 29.34 |  |
| DQ340411 | 35.04 | 15.73 | 19.83 | 29.40 |  | JN033694 | 35.24 | 16.84 | 19.99 | 27.93 |  |
| DQ340413 | 35.04 | 15.73 | 19.83 | 29.40 |  | KP715658 | 35.04 | 15.90 | 19.89 | 29.17 |  |
| DQ340404 | 35.10 | 15.73 | 19.72 | 29.46 |  | KP715661 | 35.04 | 15.90 | 19.89 | 29.17 |  |
| DQ340406 | 35.10 | 15.73 | 19.72 | 29.46 |  | KP715668 | 35.04 | 15.90 | 19.89 | 29.17 |  |
| DQ340408 | 35.16 | 15.73 | 19.66 | 29.46 |  | KP715680 | 35.04 | 15.90 | 19.89 | 29.17 |  |
| DQ340410 | 35.10 | 15.73 | 19.77 | 29.46 |  | KP715681 | 34.99 | 15.90 | 19.89 | 29.23 |  |
| KP859574 | 35.16 | 15.73 | 19.83 | 29.29 |  | KP715685 | 35.04 | 15.90 | 19.89 | 29.17 |  |
| KP859575 | 35.16 | 15.73 | 19.83 | 29.29 |  | KP715689 | 34.99 | 15.95 | 19.94 | 29.12 |  |
| KP859576 | 35.16 | 15.73 | 19.83 | 29.29 |  | KP715693 | 34.99 | 15.84 | 19.94 | 29.23 |  |
| KP859577 | 35.10 | 15.84 | 19.77 | 29.29 |  | KP715694 | 34.99 | 15.90 | 19.94 | 29.17 |  |
| KP859578 | 34.99 | 15.73 | 20.00 | 29.29 |  | KP715708 | 35.04 | 15.84 | 19.89 | 29.23 |  |
| KU983477 | 35.10 | 15.90 | 19.83 | 29.17 |  | KP715714 | 35.04 | 15.95 | 19.89 | 29.12 |  |
| KT162028 | 34.99 | 15.95 | 19.94 | 29.12 |  | KP715715 | 35.16 | 15.84 | 19.77 | 29.23 |  |
| KT162039 | 34.87 | 15.95 | 20.00 | 29.17 |  | KP715717 | 34.99 | 15.90 | 19.94 | 29.17 |  |
| KP260509 | 35.10 | 15.78 | 19.94 | 29.17 |  | GU212792 | 34.87 | 15.61 | 20.28 | 29.23 |  |
| KT162005 | 35.10 | 15.78 | 19.94 | 29.17 |  | GU212791 | 34.99 | 15.73 | 20.06 | 29.23 |  |
| KT162038 | 35.16 | 15.95 | 19.89 | 29.00 |  | GU212790 | 34.99 | 15.84 | 20.00 | 29.17 |  |
| KT382542 | 35.04 | 16.07 | 19.89 | 29.00 |  | GQ379042 | 35.04 | 15.90 | 19.89 | 29.17 |  |
| KR869652 | 34.99 | 15.95 | 19.89 | 29.17 |  | GQ379043 | 35.04 | 15.95 | 19.89 | 29.12 |  |
| KR869677 | 35.04 | 15.95 | 19.89 | 29.12 |  | GQ379044 | 34.99 | 15.90 | 19.94 | 29.17 |  |
| KR002800 | 35.04 | 15.90 | 19.89 | 29.17 |  | GQ379045 | 35.04 | 15.90 | 19.89 | 29.17 |  |
| KR002805 | 35.04 | 15.95 | 19.89 | 29.12 |  | GQ379048 | 35.04 | 15.90 | 19.89 | 29.17 |  |
| KR611459 | 34.93 | 16.01 | 20.00 | 29.06 |  | GQ379049 | 35.04 | 15.90 | 19.89 | 29.17 |  |
| KR611460 | 34.99 | 15.95 | 19.94 | 29.12 |  | KU244254 | 34.99 | 15.95 | 20.00 | 29.06 |  |
| KR611522 | 35.16 | 15.78 | 19.89 | 29.17 |  | JX048605 | 34.99 | 15.78 | 19.89 | 29.34 |  |
| KP686093 | 35.04 | 15.84 | 19.89 | 29.23 |  | JX048607 | 34.99 | 15.90 | 19.94 | 29.17 |  |
| KP749848 | 35.10 | 15.90 | 19.89 | 29.12 |  | FJ265775 | 34.99 | 15.84 | 19.83 | 29.34 |  |
| KP749849 | 35.10 | 15.90 | 19.83 | 29.17 |  | FJ265776 | 35.04 | 15.78 | 19.89 | 29.29 |  |
| KP749850 | 34.87 | 16.07 | 19.89 | 29.17 |  | FJ265782 | 34.93 | 15.95 | 19.83 | 29.29 |  |
| KP749851 | 35.04 | 15.84 | 19.94 | 29.17 |  | FJ265783 | 34.99 | 15.95 | 19.83 | 29.23 |  |
| KP749855 | 34.87 | 16.01 | 19.94 | 29.17 |  | EF592511 | 35.10 | 15.78 | 19.83 | 29.29 |  |
| KM924289 | 34.99 | 15.84 | 20.11 | 29.06 |  | AY869724 | 34.93 | 15.95 | 19.94 | 29.17 |  |
| KJ438805 | 34.99 | 15.90 | 19.94 | 29.17 |  | U72695 | 35.16 | 15.73 | 19.77 | 29.34 |  |
| KF803643 | 35.04 | 15.95 | 19.83 | 29.17 |  | U72696 | 35.10 | 15.73 | 19.83 | 29.34 |  |
| KF676668 | 35.04 | 15.95 | 19.89 | 29.12 |  | U72697 | 35.10 | 15.78 | 19.83 | 29.29 |  |
| KR058183 | 34.99 | 16.01 | 19.94 | 29.06 |  | U72698 | 34.93 | 15.78 | 19.89 | 29.40 |  |
| KR002799 | 34.99 | 15.95 | 19.94 | 29.12 |  | KJ813892 | 35.04 | 15.78 | 19.83 | 29.34 |  |
| KJ438800 | 34.99 | 15.90 | 19.89 | 29.23 |  | KJ813827 | 34.99 | 15.84 | 19.89 | 29.29 |  |
| KJ438804 | 35.04 | 15.95 | 19.83 | 29.17 |  | KJ813836 | 35.33 | 15.78 | 19.54 | 29.34 |  |
| KC262178 | 35.21 | 15.61 | 19.66 | 29.52 |  | KJ813862 | 35.10 | 15.78 | 19.89 | 29.23 |  |
| KJ438798 | 35.04 | 15.84 | 19.94 | 29.17 |  | KJ813868 | 35.16 | 15.73 | 19.83 | 29.29 |  |
| KJ438799 | 34.99 | 15.90 | 19.89 | 29.23 |  | KJ813869 | 35.27 | 15.78 | 19.77 | 29.17 |  |
| JX660690 | 35.04 | 15.90 | 19.89 | 29.17 |  | KJ813870 | 35.21 | 15.78 | 19.66 | 29.34 |  |
| JX121623 | 35.04 | 15.95 | 19.89 | 29.12 |  | KJ813876 | 34.93 | 15.90 | 19.89 | 29.29 |  |
| JX121625 | 34.93 | 15.95 | 19.94 | 29.17 |  | KJ813880 | 35.16 | 15.78 | 19.83 | 29.23 |  |
| JX121626 | 35.04 | 15.95 | 19.89 | 29.12 |  | KJ813881 | 34.87 | 15.90 | 19.94 | 29.29 |  |
| JX121627 | 34.99 | 15.90 | 19.94 | 29.17 |  | KJ813882 | 34.87 | 15.84 | 19.94 | 29.34 |  |
| JQ743901 | 34.93 | 15.95 | 19.94 | 29.17 |  | KJ813888 | 35.21 | 15.73 | 19.83 | 29.23 |  |
| JN403045 | 35.10 | 15.73 | 19.83 | 29.34 |  | JX475269 | 35.27 | 15.78 | 19.72 | 29.23 |  |
| JQ268283 | 34.99 | 15.90 | 19.89 | 29.23 |  | JX475272 | 35.16 | 15.84 | 19.83 | 29.17 |  |
| JQ268284 | 34.99 | 16.01 | 19.94 | 29.06 |  | JX475278 | 34.93 | 15.90 | 19.89 | 29.29 |  |
| JF789638 | 34.99 | 15.78 | 19.83 | 19.40 |  | JX475244 | 34.21 | 15.84 | 19.83 | 29.12 |  |
| HQ883267 | 35.04 | 16.01 | 19.89 | 29.06 |  | JX475231 | 35.21 | 15.78 | 19.66 | 29.34 |  |
| HQ651237 | 35.10 | 15.84 | 20.00 | 29.08 |  | JX475232 | 35.10 | 16.01 | 19.77 | 29.12 |  |
| JF795453 | 34.93 | 15.90 | 19.94 | 29.17 |  | JX475239 | 35.21 | 15.78 | 19.72 | 29.29 |  |
| JF795455 | 35.04 | 15.90 | 19.89 | 29.17 |  | JX475248 | 35.16 | 15.78 | 19.66 | 29.40 |  |
| JF795456 | 34.93 | 15.95 | 19.94 | 29.17 |  | JX475263 | 35.10 | 16.01 | 19.77 | 29.12 |  |
| JQ996155 | 35.10 | 15.84 | 19.77 | 29.29 |  | JX475268 | 35.21 | 15.78 | 19.66 | 29.34 |  |
| JQ996151 | 35.16 | 15.90 | 19.72 | 29.23 |  | JX475271 | 35.21 | 15.78 | 19.72 | 29.29 |  |
| JX120178 | 34.99 | 15.90 | 19.94 | 29.17 |  | JX475279 | 35.27 | 15.78 | 19.66 | 29.29 |  |
| KF638400 | 34.99 | 15.84 | 19.89 | 29.29 |  | JX475280 | 35.21 | 15.78 | 19.72 | 29.29 |  |
| JQ743891 | 34.99 | 15.90 | 19.94 | 29.17 |  | JX475286 | 35.21 | 15.78 | 19.72 | 29.29 |  |
| JQ743890 | 35.16 | 15.95 | 19.77 | 29.12 |  | JX475246 | 34.21 | 15.84 | 19.66 | 29.29 |  |
| JQ743906 | 34.87 | 16.07 | 20.00 | 29.06 |  | JX475247 | 34.93 | 15.90 | 19.89 | 29.29 |  |
| JQ743902 | 34.99 | 15.78 | 19.89 | 29.34 |  | JN867601 | 35.04 | 16.01 | 19.83 | 29.12 |  |
| KM083036 | 34.99 | 15.78 | 19.77 | 29.46 |  | JX475249 | 34.99 | 15.84 | 19.83 | 29.34 |  |
| KM083041 | 34.99 | 15.78 | 19.77 | 29.46 |  | JX475251 | 34.93 | 15.90 | 19.89 | 29.29 |  |
| KJ194462 | 35.21 | 15.61 | 19.66 | 29.52 |  | JX475260 | 35.16 | 15.78 | 19.83 | 29.23 |  |
| KC881278. | 34.99 | 15.95 | 19.94 | 29.12 |  | JX475262 | 35.21 | 15.84 | 19.83 | 29.17 |  |
| GU392239 | 35.04 | 15.73 | 19.77 | 29.46 |  | JN867606 | 35.16 | 15.84 | 19.77 | 29.23 |  |
| GU392244 | 35.04 | 15.73 | 19.77 | 29.46 |  | JX475243 | 35.16 | 15.78 | 19.83 | 29.23 |  |
| KF482468. | 34.87 | 16.01 | 20.06 | 29.06 |  | JN867599 | 35.16 | 15.73 | 19.77 | 29.34 |  |
| KF482469. | 35.04 | 15.90 | 19.89 | 29.17 |  | JN867607 | 35.21 | 15.78 | 19.77 | 29.23 |  |
| KF482470. | 35.10 | 15.78 | 19.77 | 29.34 |  | JN867602 | 34.87 | 15.84 | 19.94 | 29.34 |  |
| KF482471. | 35.10 | 15.90 | 19.83 | 29.17 |  | AY742951 | 35.04 | 15.73 | 19.89 | 29.34 |  |
| KF482476. | 35.04 | 15.78 | 19.89 | 29.29 |  | AY742955 | 34.93 | 15.90 | 19.89 | 29.29 |  |
| KF482477. | 34.99 | 15.78 | 19.89 | 29.34 |  | JX475242 | 34.99 | 15.78 | 19.94 | 29.29 |  |
| KF482478. | 35.16 | 15.84 | 19.77 | 29.23 |  | EU659119 | 35.04 | 15.73 | 19.89 | 29.34 |  |
| JF767492 | 35.04 | 15.78 | 19.83 | 29.34 |  | EU659121 | 35.04 | 15.78 | 19.94 | 29.23 |  |
| JF795452 | 34.99 | 15.95 | 19.94 | 29.12 |  | JX475240 | 35.10 | 15.78 | 19.89 | 29.23 |  |
| JF795454 | 34.87 | 16.07 | 20.00 | 29.06 |  | JX475241 | 35.10 | 15.78 | 19.89 | 29.23 |  |
| GU452713 | 35.04 | 15.78 | 19.83 | 29.34 |  | AY742936 | 35.10 | 15.78 | 19.83 | 29.29 |  |
| GU380298 | 35.04 | 15.95 | 19.89 | 29.12 |  | AY742932 | 35.04 | 15.73 | 19.83 | 29.40 |  |
| GU380305 | 35.10 | 15.90 | 19.83 | 29.17 |  | EU659118 | 35.10 | 15.90 | 19.72 | 29.29 |  |
| KJ186139 | 35.50 | 15.50 | 19.43 | 29.57 |  | EU659117 | 35.21 | 15.73 | 19.66 | 29.40 |  |
| KJ186143 | 35.04 | 15.78 | 19.83 | 29.34 |  | EU659116 | 35.21 | 15.73 | 19.66 | 29.40 |  |
| GQ857609 | 34.87 | 15.78 | 19.94 | 29.40 |  | KU508407 | 35.16 | 15.78 | 19.83 | 29.23 |  |
| GU569936 | 34.76 | 16.01 | 20.00 | 29.23 |  | JX305965 | 35.04 | 15.73 | 20.00 | 29.23 |  |
| FJ435348 | 35.33 | 15.50 | 19.60 | 29.57 |  | JX305964 | 34.99 | 15.73 | 20.00 | 29.29 |  |
| FJ435342 | 35.27 | 15.56 | 19.60 | 29.57 |  | JX305956 | 35.04 | 15.73 | 19.94 | 29.29 |  |
| FJ432717 | 35.04 | 15.95 | 19.89 | 29.12 |  | HQ025913 | 35.04 | 15.73 | 19.94 | 29.29 |  |
| EU441279 | 34.87 | 16.07 | 19.94 | 29.12 |  | JX305946 | 35.04 | 15.67 | 20.00 | 29.29 |  |
| KJ186144 | 34.87 | 16.01 | 20.00 | 29.12 |  | JX305951 | 34.99 | 15.73 | 20.00 | 29.29 |  |
| KJ186145 | 34.99 | 15.84 | 19.89 | 29.29 |  | FJ005244 | 34.99 | 15.73 | 20.00 | 29.29 |  |
| GQ857604 | 35.16 | 15.95 | 19.60 | 29.29 |  | FJ005251 | 35.10 | 15.78 | 19.89 | 29.23 |  |
| GQ857608 | 35.16 | 15.84 | 19.77 | 29.23 |  | FJ222822 | 35.04 | 15.73 | 19.89 | 29.34 |  |
| GQ857614 | 35.16 | 15.73 | 19.66 | 29.46 |  | FJ005234 | 34.99 | 15.73 | 20.00 | 29.29 |  |
| GQ169537 | 34.93 | 15.95 | 19.94 | 29.17 |  | FJ005235 | 35.16 | 15.73 | 19.83 | 29.29 |  |
| GQ169544 | 35.04 | 16.01 | 19.89 | 29.06 |  | FJ005242 | 34.99 | 15.73 | 20.00 | 29.29 |  |
| GQ169545 | 34.93 | 15.84 | 19.94 | 29.29 |  | FJ005243 | 35.21 | 15.73 | 19.77 | 29.29 |  |
| GQ169549 | 34.99 | 15.84 | 19.89 | 29.29 |  | FJ005214 | 35.10 | 15.78 | 19.89 | 29.23 |  |
| GQ169550 | 34.87 | 16.01 | 20.00 | 29.12 |  | FJ005225 | 35.16 | 15.73 | 19.83 | 29.29 |  |
| GQ169553 | 34.99 | 15.73 | 19.89 | 29.40 |  | FJ005233 | 35.10 | 15.73 | 19.89 | 29.29 |  |
| EU697385 | 35.04 | 15.95 | 19.83 | 29.17 |  | FJ005253 | 35.10 | 15.84 | 19.77 | 29.29 |  |
| EU213073 | 34.99 | 15.84 | 19.89 | 29.29 |  | FJ222824 | 35.16 | 15.67 | 19.77 | 29.40 |  |
| EU213079 | 35.04 | 15.78 | 19.83 | 29.34 |  | FJ005263 | 35.04 | 15.67 | 19.89 | 29.40 |  |
| EU170352 | 35.04 | 15.78 | 19.83 | 29.34 |  | FJ005264 | 35.10 | 15.84 | 19.77 | 29.29 |  |
| EU145953 | 35.10 | 15.78 | 19.77 | 29.34 |  | FJ005265 | 34.99 | 15.78 | 19.89 | 29.34 |  |
| EU145961 | 34.87 | 15.95 | 20.00 | 29.17 |  | FJ005213 | 35.10 | 15.73 | 19.89 | 29.29 |  |
| EF666059 | 35.10 | 15.78 | 19.77 | 29.34 |  | FJ005262 | 34.76 | 15.73 | 19.94 | 29.57 |  |
| GQ857600 | 35.10 | 15.78 | 19.72 | 29.40 |  | FJ005210 | 35.16 | 15.73 | 19.83 | 29.29 |  |
| GQ857602 | 35.16 | 15.78 | 19.72 | 29.34 |  | FJ005211 | 35.16 | 15.73 | 19.83 | 29.29 |  |
| EU377537 | 35.16 | 15.90 | 19.77 | 29.17 |  | FJ005252 | 35.10 | 15.84 | 19.77 | 29.29 |  |
| EF028071 | 34.81 | 16.30 | 19.72 | 29.17 |  | FJ005195 | 35.16 | 15.67 | 19.89 | 29.29 |  |
| DQ903936 | 34.93 | 16.24 | 19.77 | 29.06 |  | FJ222821 | 35.10 | 15.73 | 19.83 | 29.34 |  |
| EU310373 | 34.93 | 16.01 | 19.94 | 29.12 |  | FJ222823 | 34.99 | 15.67 | 19.89 | 29.46 |  |
| FJ432716 | 34.87 | 16.01 | 20.00 | 29.12 |  | KM457132 | 35.04 | 15.95 | 19.89 | 29.12 |  |
| GQ857596 | 35.16 | 15.78 | 19.72 | 29.34 |  | KM457136 | 35.04 | 15.95 | 19.89 | 29.12 |  |
| DQ177497 | 34.99 | 15.95 | 19.83 | 29.23 |  | KM457141 | 35.04 | 15.95 | 19.89 | 29.12 |  |
| DQ354068 | 35.04 | 15.78 | 19.77 | 29.40 |  | KM457143 | 35.04 | 16.01 | 19.89 | 29.06 |  |
| EF011664 | 35.10 | 15.78 | 19.77 | 29.34 |  | JF906788 | 35.04 | 15.95 | 19.89 | 29.12 |  |
| GU569942 | 35.21 | 15.78 | 19.66 | 29.34 |  | KC196088 | 35.16 | 15.73 | 19.83 | 29.29 |  |
| GU569941 | 35.10 | 15.95 | 19.77 | 29.17 |  | KC196089 | 35.21 | 15.73 | 19.83 | 29.23 |  |
| GU569937 | 35.04 | 15.84 | 19.89 | 29.23 |  | KC196093 | 35.21 | 15.73 | 19.77 | 29.29 |  |
| GU569944 | 35.16 | 15.84 | 19.83 | 29.17 |  | KM457130 | 35.16 | 15.73 | 19.83 | 29.29 |  |
| GU569946 | 35.21 | 15.73 | 19.66 | 29.40 |  | KM457131 | 35.10 | 15.78 | 19.89 | 29.23 |  |
| GU569945 | 35.10 | 15.95 | 19.77 | 29.17 |  | KC196110 | 35.04 | 15.95 | 19.89 | 29.12 |  |
| GU569947 | 35.21 | 15.78 | 19.66 | 29.34 |  | KC196114 | 34.99 | 16.01 | 19.89 | 29.12 |  |
| GU569948 | 35.16 | 15.78 | 19.66 | 29.40 |  | KC196094 | 35.04 | 15.78 | 19.94 | 29.23 |  |
| GU569943 | 35.16 | 15.61 | 19.66 | 29.46 |  | KC196098 | 35.16 | 15.78 | 19.83 | 29.23 |  |
| DQ025974 | 35.16 | 15.73 | 19.83 | 29.29 |  | KM457102 | 35.04 | 15.95 | 19.89 | 29.12 |  |
| DQ025981 | 35.21 | 15.73 | 19.77 | 29.29 |  | KM457119 | 35.16 | 15.78 | 19.83 | 29.23 |  |
| DQ025982 | 35.04 | 15.78 | 19.83 | 29.34 |  | KM457120 | 35.21 | 15.67 | 19.77 | 29.34 |  |
| DQ025985 | 35.10 | 15.78 | 19.89 | 29.23 |  | KM457122 | 35.21 | 15.73 | 19.77 | 29.29 |  |
| DQ025990 | 35.10 | 15.78 | 19.83 | 29.29 |  | KC196079 | 35.10 | 15.84 | 19.89 | 29.17 |  |
| DQ025991 | 34.93 | 15.84 | 19.89 | 29.34 |  | KC196100 | 35.10 | 15.78 | 19.89 | 29.23 |  |
| DQ025992 | 34.93 | 15.73 | 19.89 | 29.46 |  | KC196101 | 35.10 | 15.78 | 19.89 | 29.23 |  |
| DQ025993 | 34.99 | 15.84 | 19.94 | 29.23 |  | KC196102 | 35.10 | 15.78 | 19.89 | 29.23 |  |
| DQ025994 | 35.10 | 15.73 | 19.89 | 29.29 |  | KM457115 | 35.10 | 15.95 | 19.89 | 29.06 |  |
| DQ025995 | 35.04 | 15.78 | 19.83 | 29.34 |  | KM457116 | 35.10 | 15.78 | 19.83 | 29.29 |  |
| DQ026002 | 35.10 | 15.78 | 19.77 | 29.34 |  | KM457118 | 35.21 | 15.73 | 19.77 | 29.29 |  |
| DQ025951 | 35.16 | 15.78 | 19.83 | 29.23 |  | KC196103 | 35.10 | 15.95 | 19.89 | 29.06 |  |
| DQ025956 | 35.10 | 15.67 | 19.83 | 29.40 |  | KC196104 | 35.16 | 15.84 | 19.89 | 29.12 |  |
| DQ025957 | 34.99 | 15.78 | 19.89 | 29.34 |  | KC196109 | 35.16 | 15.73 | 19.83 | 29.29 |  |
| DQ025960 | 35.16 | 15.78 | 19.83 | 29.23 |  | KM457111 | 35.16 | 15.73 | 19.83 | 29.29 |  |
| DQ025961 | 34.99 | 15.84 | 19.83 | 29.34 |  | KM457113 | 35.10 | 15.78 | 19.89 | 29.23 |  |
| DQ025963 | 34.99 | 15.84 | 19.89 | 29.29 |  | KM457114 | 35.16 | 15.84 | 19.89 | 29.12 |  |
| DQ025966 | 35.16 | 15.73 | 19.83 | 29.29 |  | KC196085 | 35.21 | 15.73 | 19.83 | 29.23 |  |
| DQ025967 | 34.99 | 15.78 | 19.89 | 29.34 |  | KM457107 | 35.16 | 15.73 | 19.83 | 29.29 |  |
| DQ025968 | 35.16 | 15.73 | 19.83 | 29.29 |  | KM457108 | 35.21 | 15.78 | 19.77 | 29.23 |  |
| DQ025944 | 35.04 | 15.78 | 19.83 | 29.34 |  | KM457110 | 35.10 | 15.78 | 19.89 | 29.23 |  |
| DQ025945 | 34.99 | 15.78 | 19.89 | 29.34 |  | KC196086 | 35.16 | 15.73 | 19.83 | 29.29 |  |
| DQ025950 | 34.99 | 15.78 | 19.89 | 29.34 |  | KC196087 | 35.27 | 15.78 | 19.72 | 29.23 |  |
| DQ025942 | 35.16 | 15.73 | 19.83 | 29.29 |  | KC196099 | 35.16 | 15.84 | 19.89 | 29.12 |  |
| DQ025943 | 34.99 | 15.84 | 19.89 | 29.29 |  | KM457103 | 35.04 | 15.78 | 19.89 | 29.29 |  |
| FJ005204 | 35.16 | 15.73 | 19.83 | 29.29 |  | KM457105 | 35.27 | 15.78 | 19.72 | 29.23 |  |
| FJ005202 | 35.16 | 15.78 | 19.83 | 29.23 |  | KM457106 | 35.16 | 15.73 | 19.83 | 29.29 |  |
| FJ005203 | 35.16 | 15.73 | 19.83 | 29.29 |  | KF149962 | 35.10 | 15.78 | 19.89 | 29.23 |  |
| FJ005260 | 35.10 | 15.78 | 19.83 | 29.29 |  | KF149965 | 35.21 | 15.78 | 19.77 | 29.23 |  |
| FJ005261 | 35.16 | 15.78 | 19.66 | 29.40 |  | KF149967 | 35.10 | 15.78 | 19.89 | 29.23 |  |
| FJ005196 | 35.16 | 15.78 | 19.83 | 29.23 |  | KF149971 | 35.04 | 15.78 | 19.89 | 29.29 |  |
| FJ005197 | 35.16 | 15.73 | 19.83 | 29.29 |  | KF149972 | 34.93 | 15.84 | 19.89 | 29.34 |  |
| FJ005201 | 35.16 | 15.78 | 19.83 | 29.23 |  | KF149973 | 35.04 | 15.78 | 19.83 | 29.34 |  |
| AY742934 | 34.99 | 15.78 | 19.89 | 29.34 |  | KF149975 | 35.04 | 15.78 | 19.83 | 29.34 |  |
| AY742935 | 34.99 | 15.78 | 19.89 | 29.34 |  | KF149976 | 35.04 | 15.78 | 19.83 | 29.34 |  |
| JN625219 | 35.21 | 15.78 | 19.72 | 29.29 |  | KF149979 | 35.04 | 15.78 | 19.83 | 29.34 |  |
| JN625220 | 35.21 | 15.90 | 19.60 | 29.29 |  | KF149984 | 35.21 | 15.73 | 19.77 | 29.29 |  |
| JN625221 | 35.16 | 15.73 | 19.77 | 29.34 |  | KF149985 | 34.99 | 15.90 | 19.89 | 29.23 |  |
| JN625222 | 35.16 | 15.67 | 19.83 | 29.34 |  | Mean | 34.98 | 15.79 | 19.80 | 29.15 |  |
| JN625223 | 35.04 | 15.95 | 19.94 | 29.06 |  | SD | 0.118 | 0.113 | 0.093 | 0.66 |  |
| **JN625224** | **35.04** | **15.61** | **19.89** | **29.46** |  |  |  |  |  |  |  |
| KF366250 | 35.04 | 15.95 | 19.94 | 29.06 |  |  |  |  |  |  |  |

**Table S3 The correlation analysis of codon usage indices.**

|  | ***A*** | ***C*** | ***G*** | ***T*** | ***GC*** | ***1st axis*** | ***2nd axis*** | ***Gravy*** | ***Aroma*** |
| --- | --- | --- | --- | --- | --- | --- | --- | --- | --- |
| ***T3s*** | 0.216** | 0.784** | -0.471** | 0.159** | 0.116* | -0.339** | 0.180** | -0.109* | 0.297** |
| ***C3s*** | -0.370** | 0.890** | 0.379** | -0.188** | 0.113* | 0.442** | -0.091 | 0.128** | -0.406** |
| ***A3s*** | 0.655** | 0.525** | -0.584** | 0.093 | -0.071 | -0.460** | -0.059 | -0.172** | 0.313** |
| ***G3s*** | -0.536** | 0.388** | 0.677** | -0.050 | 0.058 | 0.431** | -0.059 | 0.142** | -0.120* |
| ***ENC*** | -0.509** | 0.688** | 0.487** | -0.159** | 0.096* | 0.802** | -0.046 | 0.192** | -0.240** |
| ***GC3s*** | -0.496** | 0.559* | 0.551** | -0.101* | 0.124* | 0.390** | 0.092 | 0.158** | -0.268** |
|  |  |  |  |  |  |  |  |  |  |

*Signifies 0.05>p>0.01； **signifies p<0.01

**Table S4 The abundance of the 16 dinucleotides.**

| **AA** | **AC** | **AG** | **AT** | **CA** | **CC** | **CG** | **CT** |
| --- | --- | --- | --- | --- | --- | --- | --- |
| 0.98 | 1.182 | 0.865 | 1.013 | 1.37 | 1.139 | 0.22 | 1.012 |
| **GA** | **GC** | **GG** | **GT** | **TA** | **TC** | **TG** | **TT** |
| 0.933 | 0.959 | 1.432 | 0.811 | 0.865 | 0.737 | 1.293 | 1.108 |

**Table S5 The detail information of the 424 sequences.**

| **Number** | **location** | **year** | **Number** | **location** | **year** |
| --- | --- | --- | --- | --- | --- |
| KM236573 | Argentina | 2012 | FJ005196 | Germany | 1997 |
| KM236570 | Argentina | 2012 | FJ005197 | Germany | 1997 |
| KM236569 | Argentina | 2012 | FJ005201 | Germany | 1997 |
| KM236568 | Argentina | 2012 | FJ005202 | Germany | 1998 |
| JF414822 | Argentina | 2010 | FJ005203 | Germany | 1998 |
| JF414825 | Argentina | 2010 | FJ005204 | Germany | 1999 |
| JF414826 | Argentina | 2010 | GQ865519 | Greece | 2009 |
| JF414823 | Argentina | 2009 | GQ865518 | Greece | 2008 |
| JF414820 | Argentina | 2009 | KF539789 | Hungary | 2012 |
| JF414821 | Argentina | 2009 | KF539790 | Hungary | 2012 |
| JF414818 | Argentina | 2008 | KF539791 | Hungary | 2012 |
| JF414819 | Argentina | 2008 | KF539792 | Hungary | 2012 |
| JF346754 | Argentina | 2003 | KF539797 | Hungary | 2012 |
| JF414817 | Argentina | 2003 | KF539802 | Hungary | 2012 |
| KU508691 | Australia | 2015 | KF539803 | Hungary | 2012 |
| KU508692 | Australian | 2015 | KF539804 | Hungary | 2012 |
| KU508693 | Australian | 2015 | KF539805 | Hungary | 2012 |
| KT275255 | Portugal | 2014 | JN625219 | India | 2011 |
| KT275256 | Portugal | 2014 | JN625220 | India | 2011 |
| KR559896 | Portugal | 2014 | JN625221 | India | 2011 |
| KT275254 | Portugal | 2013 | JN625222 | India | 2011 |
| KR559895 | Portugal | 2013 | JN625223 | India | 2011 |
| KT275252 | Portugal | 2012 | JN625224 | India | 2011 |
| KT275253 | Portugal | 2012 | KF366250 | India | 2013 |
| KR559891 | Portugal | 2012 | AB120720 | Japan | 2003 |
| KR559892 | Portugal | 2012 | AB120723 | Japan | 2003 |
| KR559893 | Portugal | 2012 | AB120724 | Japan | 2003 |
| KR559894 | Portugal | 2012 | AB120728 | Japan | 2003 |
| DQ340404 | Brazil | 1980 | AB128923 | Japan | 2003 |
| DQ340406 | Brazil | 1980 | AB115504 | Japan | 2003 |
| DQ340408 | Brazil | 1980 | AB054214 | Japan | 2001 |
| DQ340410 | Brazil | 1980 | AB054220 | Japan | 2001 |
| DQ340411 | Brazil | 1990 | AB054221 | Japan | 2001 |
| DQ340413 | Brazil | 1990 | AB054222 | Japan | 2001 |
| DQ340415 | Brazil | 1991 | AB054223 | Japan | 2001 |
| DQ340417 | Brazil | 1991 | AB054224 | Japan | 2001 |
| DQ340419 | Brazil | 1992 | D78585 | Japan | 1995 |
| DQ340420 | Brazil | 1992 | KP893077 | South Korea | 2014 |
| DQ340421 | Brazil | 1992 | KP893078 | South Korea | 2014 |
| DQ340422 | Brazil | 1993 | EF599098 | South Korea | 2007 |
| DQ340425 | Brazil | 1994 | EU009206 | South Korea | 2007 |
| DQ340426 | Brazil | 1994 | EU009205 | South Korea | 2007 |
| DQ340428 | Brazil | 1994 | EU009200 | South Korea | 2007 |
| DQ340429 | Brazil | 1994 | EF599096 | South Korea | 2007 |
| DQ340430 | Brazil | 1995 | FJ197823 | South Korea | 2007 |
| DQ340432 | Brazil | 1995 | FJ197830 | South Korea | 2007 |
| DQ340433 | Brazil | 2000 | FJ197831 | South Korea | 2007 |
| DQ340434 | Brazil | 2000 | FJ197832 | South Korea | 2007 |
| KP859574 | Croatia | 2014 | FJ197839 | South Korea | 2007 |
| KP859575 | Croatia | 2014 | FJ197840 | South Korea | 2007 |
| KP859576 | Croatia | 2014 | FJ197841 | South Korea | 2007 |
| KP859577 | Croatia | 2014 | FJ197842 | South Korea | 2007 |
| KP859578 | Croatia | 2014 | FJ197845 | South Korea | 2007 |
| KU983477 | China | 2016 | EF189717 | South Korea | 2006 |
| KT162028 | China | 2015 | AY742933 | New Zealand | 1993 |
| KT162039 | China | 2015 | JN033694 | Russia | 1993 |
| KP260509 | China | 2014 | KP715658 | Thailand | 2014 |
| KT162005 | China | 2014 | KP715661 | Thailand | 2014 |
| KT162038 | China | 2014 | KP715668 | Thailand | 2014 |
| KT382542 | China | 2014 | KP715680 | Thailand | 2014 |
| KR869652 | China | 2014 | KP715681 | Thailand | 2014 |
| KR869677 | China | 2014 | KP715685 | Thailand | 2014 |
| KR002800 | China | 2014 | KP715689 | Thailand | 2014 |
| KR002805 | China | 2014 | KP715693 | Thailand | 2014 |
| KR611459 | China | 2014 | KP715694 | Thailand | 2014 |
| KR611460 | China | 2014 | KP715708 | Thailand | 2014 |
| KR611522 | China | 2014 | KP715714 | Thailand | 2014 |
| KP686093 | China | 2014 | KP715715 | Thailand | 2014 |
| KP749848 | China | 2014 | KP715717 | Thailand | 2014 |
| KP749849 | China | 2014 | GU212792 | Thailand | 2009 |
| KP749850 | China | 2014 | GU212791 | Thailand | 2009 |
| KP749851 | China | 2014 | GU212790 | Thailand | 2009 |
| KP749855 | China | 2014 | GQ379042 | Thailand | 2008 |
| KM924289 | China | 2013 | GQ379043 | Thailand | 2008 |
| KJ438805 | China | 2013 | GQ379044 | Thailand | 2009 |
| KF803643 | China | 2013 | GQ379045 | Thailand | 2009 |
| KF676668 | China | 2013 | GQ379048 | Thailand | 2008 |
| KR058183 | China | 2013 | GQ379049 | Thailand | 2008 |
| KR002799 | China | 2013 | JX048605 | Taiwan | 2011 |
| KJ438800 | China | 2012 | JX048607 | Taiwan | 2011 |
| KJ438804 | China | 2012 | FJ265775 | Taiwan | 2008 |
| KC262178 | China | 2012 | FJ265776 | Taiwan | 2008 |
| KJ438798 | China | 2011 | FJ265782 | Taiwan | 2008 |
| KJ438799 | China | 2011 | FJ265783 | Taiwan | 2008 |
| JX660690 | China | 2011 | EF592511 | Taiwan | 2006 |
| JX121623 | China | 2011 | AY869724 | Taiwan | 2004 |
| JX121625 | China | 2011 | U72695 | Taiwan | 1996 |
| JX121626 | China | 2011 | U72696 | Taiwan | 1996 |
| JX121627 | China | 2011 | U72697 | Taiwan | 1996 |
| JQ743901 | China | 2011 | U72698 | Taiwan | 1996 |
| JN403045 | China | 2011 | KU244254 | Taiwan | 2015 |
| JQ268283 | China | 2011 | KJ813827 | USA | 2013 |
| JQ268284 | China | 2011 | KJ813836 | USA | 2013 |
| JF789638 | China | 2010 | KJ813862 | USA | 2013 |
| HQ883267 | China | 2010 | KJ813868 | USA | 2013 |
| HQ651237 | China | 2010 | KJ813869 | USA | 2013 |
| JF795453 | China | 2010 | KJ813870 | USA | 2013 |
| JF795455 | China | 2010 | KJ813876 | USA | 2012 |
| JF795456 | China | 2010 | KJ813880 | USA | 2012 |
| JQ996155 | China | 2010 | KJ813881 | USA | 2012 |
| JQ996151 | China | 2010 | KJ813882 | USA | 2012 |
| JX120178 | China | 2010 | KJ813888 | USA | 2012 |
| KF638400 | China | 2010 | KJ813892 | USA | 2013 |
| JQ743891 | China | 2010 | JX475231 | USA | 2011 |
| JQ743890 | China | 2010 | JX475232 | USA | 2011 |
| JQ743906 | China | 2010 | JX475239 | USA | 2011 |
| JQ743902 | China | 2010 | JX475240 | USA | 1999 |
| KM083036 | China | 2010 | JX475241 | USA | 1999 |
| KM083041 | China | 2010 | JX475242 | USA | 2002 |
| KJ194462 | China | 2010 | JX475243 | USA | 2009 |
| KC881278 | China | 2010 | JX475244 | USA | 2011 |
| GU392239 | China | 2009 | JX475246 | USA | 2010 |
| GU392244 | China | 2009 | JX475247 | USA | 2010 |
| KF482468 | China | 2009 | JX475248 | USA | 2011 |
| KF482469 | China | 2009 | JX475249 | USA | 2010 |
| KF482470 | China | 2009 | JX475251 | USA | 2009 |
| KF482471 | China | 2009 | JX475260 | USA | 2010 |
| KF482476 | China | 2009 | JX475262 | USA | 2010 |
| KF482477 | China | 2009 | JX475263 | USA | 2011 |
| KF482478 | China | 2009 | JX475268 | USA | 2011 |
| JF767492 | China | 2009 | JX475269 | USA | 2012 |
| JF795452 | China | 2009 | JX475271 | USA | 2011 |
| JF795454 | China | 2009 | JX475272 | USA | 2012 |
| GU452713 | China | 2009 | JX475278 | USA | 2012 |
| GU380298 | China | 2009 | JX475279 | USA | 2011 |
| GU380305 | China | 2009 | JX475280 | USA | 2011 |
| KJ186139 | China | 2008 | JX475286 | USA | 2011 |
| KJ186143 | China | 2008 | JN867607 | USA | 2008 |
| GQ857609 | China | 2008 | JN867606 | USA | 2009 |
| GU569936 | China | 2008 | JN867602 | USA | 2008 |
| FJ435348 | China | 2008 | JN867601 | USA | 2010 |
| FJ435342 | China | 2008 | JN867599 | USA | 2009 |
| FJ432717 | China | 2008 | EU659116 | USA | 1979 |
| EU441279 | China | 2008 | EU659117 | USA | 1980 |
| KJ186144 | China | 2007 | EU659118 | USA | 1981 |
| KJ186145 | China | 2007 | EU659119 | USA | 2000 |
| GQ857604 | China | 2007 | EU659121 | USA | 1999 |
| GQ857608 | China | 2007 | AY742936 | USA | 1998 |
| GQ857614 | China | 2007 | AY742951 | USA | 2003 |
| GQ169537 | China | 2006-2008 | AY742955 | USA | 2003 |
| GQ169544 | China | 2006-2008 | AY742932 | USA | 1991 |
| GQ169545 | China | 2006-2008 | JX305946 | Italy | 2009 |
| GQ169549 | China | 2006-2008 | JX305951 | Italy | 2009 |
| GQ169550 | China | 2006-2008 | JX305956 | Italy | 2010 |
| GQ169553 | China | 2006-2008 | JX305964 | Italy | 2011 |
| EU697385 | China | 2007 | JX305965 | Italy | 2012 |
| EU213073 | China | 2007 | KU508407 | Italy | 2016 |
| EU213079 | China | 2007 | HQ025913 | Italy | 2010 |
| EU170352 | China | 2007 | FJ222824 | Italy | 2005 |
| EU145953 | China | 2007 | FJ005262 | Italy | 2004 |
| EU145961 | China | 2007 | FJ005263 | Italy | 2005 |
| EF666059 | China | 2007 | FJ005264 | Italy | 2005 |
| GQ857600 | China | 2006 | FJ005265 | Italy | 2005 |
| GQ857602 | China | 2006 | FJ222821 | Italy | 2000 |
| EU377537 | China | 2006 | FJ222822 | Italy | 2008 |
| EF028071 | China | 2006 | FJ222823 | Italy | 1997 |
| DQ903936 | China | 2006 | FJ005210 | Italy | 2004 |
| EU310373 | China | 2006 | FJ005211 | Italy | 2004 |
| FJ432716 | China | 2006 | FJ005213 | Italy | 2005 |
| GQ857596 | China | 2005 | FJ005214 | Italy | 2006 |
| DQ177497 | China | 2005 | FJ005225 | Italy | 2006 |
| DQ354068 | China | 2004 | FJ005233 | Italy | 2006 |
| EF011664 | China | 2004 | FJ005234 | Italy | 2007 |
| GU569942 | China | 2002 | FJ005235 | Italy | 2007 |
| GU569941 | China | 2002 | FJ005242 | Italy | 2007 |
| GU569937 | China | 2002 | FJ005243 | Italy | 2007 |
| GU569944 | China | 2002 | FJ005244 | Italy | 2008 |
| GU569946 | China | 2001 | FJ005251 | Italy | 2008 |
| GU569945 | China | 2001 | FJ005252 | Italy | 2002 |
| GU569947 | China | 1999 | FJ005253 | Italy | 2005 |
| GU569948 | China | 1986 | FJ005195 | Italy | 2000 |
| GU569943 | China | 1983 | KC196079 | Uruguay | 2009 |
| KF149962 | Ecuador | 2012 | KC196085 | Uruguay | 2007 |
| KF149965 | Ecuador | 2012 | KC196086 | Uruguay | 2006 |
| KF149967 | Ecuador | 2012 | KC196087 | Uruguay | 2006 |
| KF149971 | Ecuador | 2012 | KC196088 | Uruguay | 2011 |
| KF149972 | Ecuador | 2012 | KC196089 | Uruguay | 2011 |
| KF149973 | Ecuador | 2012 | KC196093 | Uruguay | 2011 |
| KF149975 | Ecuador | 2012 | KC196094 | Uruguay | 2010 |
| KF149976 | Ecuador | 2012 | KC196098 | Uruguay | 2010 |
| KF149979 | Ecuador | 2012 | KC196099 | Uruguay | 2006 |
| KF149984 | Ecuador | 2012 | KC196100 | Uruguay | 2009 |
| KF149985 | Ecuador | 2012 | KC196101 | Uruguay | 2009 |
| DQ025942 | France | 2001 | KC196102 | Uruguay | 2009 |
| DQ025943 | France | 2001 | KC196103 | Uruguay | 2008 |
| DQ025944 | France | 2002 | KC196104 | Uruguay | 2008 |
| DQ025945 | France | 2002 | KC196109 | Uruguay | 2008 |
| DQ025950 | France | 2002 | KC196110 | Uruguay | 2011 |
| DQ025951 | France | 2003 | KC196114 | Uruguay | 2011 |
| DQ025956 | France | 2003 | KM457102 | Uruguay | 2010 |
| DQ025957 | France | 2003 | KM457103 | Uruguay | 2006 |
| DQ025960 | France | 2003 | KM457105 | Uruguay | 2006 |
| DQ025961 | France | 2003 | KM457106 | Uruguay | 2006 |
| DQ025963 | France | 2003 | KM457107 | Uruguay | 2007 |
| DQ025966 | France | 2003 | KM457108 | Uruguay | 2007 |
| DQ025967 | France | 2003 | KM457110 | Uruguay | 2007 |
| DQ025968 | France | 2003 | KM457111 | Uruguay | 2008 |
| DQ025974 | France | 2004 | KM457113 | Uruguay | 2008 |
| DQ025981 | France | 2004 | KM457114 | Uruguay | 2008 |
| DQ025982 | France | 2004 | KM457115 | Uruguay | 2009 |
| DQ025985 | France | 2004 | KM457116 | Uruguay | 2009 |
| DQ025990 | France | 2004 | KM457118 | Uruguay | 2009 |
| DQ025991 | France | 2004 | KM457119 | Uruguay | 2010 |
| DQ025992 | France | 2004 | KM457120 | Uruguay | 2010 |
| DQ025993 | France | 2004 | KM457122 | Uruguay | 2010 |
| DQ025994 | France | 2004 | KM457130 | Uruguay | 2011 |
| DQ025995 | France | 2004 | KM457131 | Uruguay | 2011 |
| DQ026002 | France | 2004 | KM457132 | Uruguay | 2010 |
| AY742934 | Germany | 1995 | KM457136 | Uruguay | 2011 |
| AY742935 | Germany | 1995 | KM457141 | Uruguay | 2011 |
| FJ005260 | Germany | 1997 | KM457143 | Uruguay | 2011 |
| FJ005261 | Germany | 1997 | JF906788 | Uruguay | 2011 |
|  |  |  |  |  |  |
